# Supplementary material for: Non-communicable diseases risk factors among the forcefully displaced Rohingya population in Bangladesh
Source: PLOS Glob Public Health. 2022 Sep 27;2(9):e0000930. doi: 10.1371/journal.pgph.0000930 (PMC10022334; doi:10.1371/journal.pgph.0000930)
Supplement: S1 File — (PDF) [file pgph.0000930.s001.pdf]

To whom it may concern

Subject: Clearance from Ethical Review Committee (ERC)

This is to certify that **Dr Ayesha Rahman (ID#17-90382-3)** enrolled at the Masters in Public Health (MPH) Program under the Department of Public Health in the American International University-Bangladesh (AIUB). She has presented a research proposal on 16 November 2018 entitled **"Prevalence of NCD risk factors and diabetes among the newly exiled Rohingya refugees."** to the Research Review Committee (RRC) and Ethical Review Committee (ERC) under the Department of Public Health. Both the Committees examined the research proposal carefully and approved for undertaking research work for the completion of dissertation which is a pre-requisite for obtaining the degree of MPH from AIUB.

The Ethical Review Committee (ERC) examined the scientific and social implication of the research proposal and ethical aspects of research involving human subjects under social context. The elements the committee examined include the justification of research, moral imperatives, ethical principles and the laws. The Proposal fulfills two essential components:

A. The selection and achievement of morally acceptable ends and
B. The morally acceptable means to those ends.

Guiding ethical principles express common standards, values and aspirations which the researchers promised to follow during different phases of the study. They are as follows:

1. Respect for human dignity.
2. Respect for free and informed consent.
3. Respect for vulnerable persons.
4. Respect for privacy and confidentiality.
5. Respect for justice and inclusiveness.
6. Balancing harms and benefits.
7. Minimizing harm and
8. Maximizing benefits.

With the conditions mentioned above the ERC approved the above noted study which consists of the articulation of national norms that are applied through prospective ethics and review of research projects.

Professor Ahmad A. N. Neaz, DSSc.
Advisor and Head, Department of Public Health,
American International University-Bangladesh (AIUB)

November 23, 2018
