# Supplementary material for: Non-communicable diseases risk factors among the forcefully displaced Rohingya population in Bangladesh
Source: PLOS Glob Public Health. 2022 Sep 27;2(9):e0000930. doi: 10.1371/journal.pgph.0000930 (PMC10022334; doi:10.1371/journal.pgph.0000930)
Supplement: Attachment — Submitted filename: Response to reviewers.docx [file pgph.0000930.s002.docx]

**Non-communicable diseases risk factors among the forcefully displaced Rohingya population in Bangladesh (Manuscript ID: PGPH-D-22-00771)**

**Journal Requirements:**

Please review your reference list to ensure that it is complete and correct. If you have cited papers that have beenretracted, please include the rationale for doing so in the manuscript text, or remove these references and replace them withrelevant current references. Any changes to the reference list should be mentioned in the rebuttal letter that accompaniesyour revised manuscript. If you need to cite a retracted article, indicate the article’s retracted status in the References list andalso include a citation and full reference for the retraction notice.

**Reply:** Thank you. We reviewed the reference list. We found one reference retracted (reference no 5) which we replaced with a relevant and current reference. We also removed one reference (reference number 19) and replaced it with a more relevant one (reference number 23). Moreover, we added seven new references which have been mentioned below along with the respective line numbers.

**Retracted reference:**

5. King, R., 2001. International Organization for Migration, World Migration Report 2000. JOURNAL OF ETHNIC AND MIGRATION STUDIES, 27(3), pp.553-553.

**Replaced reference:**

5. Kim, H.C. and Oh, S.M., 2013. Non-communicable diseases: current status of major modifiable risk factors in Korea. Journal of preventive medicine and public health, 46(4), p.165.

**Removed reference:**

19. Shi, L.L., Bradford, E., Depalo, D.E. and Chen, A.Y., 2019. Betel quid use and Oral Cancer in a high-risk refugee community in the USA: the effectiveness of an awareness initiative. Journal of Cancer Education, 34(2), pp.309-314.

**Replaced reference :**

23. Jawad, M., Khader, A. and Millett, C., 2016. Differences in tobacco smoking prevalence and frequency between adolescent Palestine refugee and non-refugee populations in Jordan, Lebanon, Syria, and the West Bank: cross-sectional analysis of the Global Youth Tobacco Survey. Conflict and health, 10(1), pp.1-8.

**Additional Editor’s comments to the authors-**

**Comment**: Please carefully address the comments and concerns raised by the reviewers. Additionally, justify and discuss why and how a convenience sample serves the purpose of adequately addressing the study aim. In the discussion section provide a comparison of the study findings with extant literature on refugee health and NCD risk factors among refugees.

**Reply:** Thank you for your comments. We have revised the whole manuscript. We have considered and incorporated the revisions suggested by the reviewers. We have also addressed the issue regarding the convenience sampling method and discussed it in the comment sections. We have provided comparisons of the study findings with extant literature on refugee health and NCD risk factors in the discussion section.We highly appreciate your suggestion and comment.

**Reviewer 1’s comments to the authors-**

**Comment 1:**  language and grammar edit are essential for this manuscript. For example, using the past tense in the methods and results section is an essential part of a scientific presentation in an article.

**Reply 1:** Thank you. Language and grammar have been revised and changed in the revised manuscript.

**Comment 2**: Abstract, results, lines 38-44: the prepared part of the results in the abstract should reflect the results of the steps of the STEPS survey appropriately to make this part more informative.

**Reply 2:** Thank you. The prepared part of the results in the abstract has been re- arranged as per the steps of the STEPS survey.

**Changes in the text:**

**Line 39-42**: In general, the findings of NCD risk factors as per STEPS survey was 53.4% for smoking and smokeless tobacco, 2.8% for alcohol consumption, 23.7% for inadequate vegetable and fruit intake, 34.5% for taking extra salt, 89.6% for insufficient physical activity, 44.5% for confirmed hypertension, 16.9% for overweight 1.2% for obesity and 0.8% for high blood sugar.

**Comment 3:** Introduction: a focus on modifiable risk factors of NCDs could be beneficial in this section.

**Reply 3:** Thank you. We appreciate your suggestion. A focus on modifiable risk factors has been added in the introduction.

**Changes in the text:**

**Lines 55-59**: Tobacco use, physical inactivity, unhealthy diet and harmful use of alcohol are considered to be the modifiable risk factors of NCDs that can be prevented by applying prior interventions [3-4].In recent times, global health policymakers are more concerned about the importance of the timely prevention, detection and correction of these modifiable risk factors to reduce the overall NCD mortality [5].

**Comment 4:** Methods, lines 94-104: the sampling method used for this seems to be a limitation and major issue needing explanation.How did authors made sure about including an un-biased sample of participants in this study?

**Reply 3:** Thank you**.** The issue has been addressed in the following lines.

**Lines 282-290:** The sampling method we used for the study is convenient sampling. We conducted the study among refugees from two pre-selected camps who voluntarily agreed to participate in the study. Since the law enforcement authority of government of Bangladesh maintains a strict surveillance system over the refugees we faced difficulty in conducting a broader study over them including every refugee settlement. In addition, we found many of the refugees who experienced various trauma and persecution from the forceful migration not intent enough to participate in the interviews. This is why we preferred convenient sampling as a method which we understand not to be completely unbiased.

**Comment 5**: Methods, lines 105-110: since the authors used the Bangla version of the STEPS questionnaire for this study and no adapted questionnaire was developed and implemented for the refugee population, it is a major limitation of this study and authors may expand in this issue in the limitation of the study.

**Reply 5**: Thank you. We have discussed about the issue in the following lines.

**Lines 114-120:** An adapted (mostly for socio-demographic background) questionnaire for this study was developed using step-I, step-II and, steps III of WHO STEPS protocol based on the 2010 STEPS survey in Bangladesh [14]. Bangla version of the STEPS questionnaire was orally translated into the local Rohingya language by an interpreter during data collection. Although the Rohingya refugees originally live in Mayanmar, the language they speak is similar to Cox’s bazar’s local dialect which assisted the local medical assistants to conduct the interviews.

**Comment 6:** Methods, lines 111-115**:** a separate part is needed in the methods section entitled study variables and the part should explain all variables used in the three steps of study and with appropriate referencing. Also, lines 121-130 could be moved to this section. Also, referring the readers to the supplementary files could be helpful in this regard.

**Reply 6:** Thank you. We appreciate your review. The issue has been addressed in the following lines.

**Lines127-154:**

**Ascertainment of the key variables:**

**Socio-demographic and behavioral variables (Step I):** Only the core questions about demographic information (step I) with few omissions such as date of birth (due to lack of accuracy on the part of the refugees), marital status etc were used to simplify the questionnaire for the limited time setting. The behavioral components (step I) included the core questions on tobacco, alcohol, physical activity, fruit and vegetable intake and extra salt intake. Information on previous history and treatment of hypertension were also obtained. The average time spent on moderate and vigorous physical activity was transformed into minutes per week. Physical activity less than 150 minutes per week was considered low. A standard measuring cup was used to obtain information on serving sizes of fruits and vegetables in a week.

**Physical and Biochemical variables (Step II and III):** In step II, the physical measurements i.e height, weight and blood pressure were measured. The biochemical measurement in step III only included random blood sugar measurement. Systolic and diastolic blood pressure was measured using a manual aneroid sphygmomanometer with an average-sized cuff at sitting and lying positions. The average of the two measurements was used for analysis. Height was recorded in centimeters, and weight was recorded in kilograms using a portable digital weighing scale. Random blood sugar was measured using a standard glucometer.

According to the American Diabetes Association (ADA) guideline, the reference values of random blood sugar between 4.4-7.8 mmol/dl was considered normal, 7.8-11.1 mmol/dl was considered pre-diabetes, and ≥ 11.1 mmol/dl was considered diabetes.

According to the Seventh Joint National Committee (JNC 7), systolic blood pressure <120 mmHg was considered as normal, 120-139 mmHg as pre-hypertension, 140-159 mmHg stage I hypertension, and ≥160 mmHg considered stage II hypertension. Diastolic blood pressure ≤80 mmHg was considered normal, 80-89 mmHg as pre-hypertension, 90-99and ≥90 mmHg as stage I hypertension and ≥100 considered as stage II hypertension.

According to the WHO guideline 2020, Body Mass Index (BMI) was classified as ≤18.5 as underweight, 18.5-24.99 as normal, 25-29.9 as pre-obesity, and ≥30 as Obesity (class I).

**Comment 7:** Methods:the WHO STEPS survey includes much more physical measurements and laboratory tests in steps two and three. However, these steps were much briefer in this study. This issue needs explanation by authors.

**Reply 7**: Thank you. In our study, we have followed an adapted questionnaire from the STEPS survey done in 2010 which was much briefer than the latest version of the STEPS survey. Considering the vulnerability of the study population we preferred to adopt the briefer version of the STEPS survey.

**Comment 8**. Methods, data analysis: the method of calculating the prevalence values and 95% confidence intervals should be provided in this part of the methods section.

**Reply 8:** Thank you. In our study we have only calculated the proportions of the NCD risk factors. The confidence interval is seen to see the distribution among the population. The issue has been addressed in the following lines.

**Lines 155-160**: The data was at first entered in Microsoft Excel 2010, and after editing and logical checking; it was analyzed in SPSS version 22.0. Descriptive analysis like frequency, percentage, mean and standard deviation were done as appropriate for the categorical variables. Chi-square test and student t-test were performed to see the association among the categories setting the α level at 0.05. 95% confidence interval was done to see the distribution among the population.

**Comment 9.** Results, line 139: the term “gender” should be replaced with “sex” since these two terms are technically different and could not be used interchangeably.

**Reply 9**: Thank you. We have replaced the word accordingly.

**Comment 10**: Results: adding some figures for data presentation in some parts of this section is suggested

**Reply 10:** Thank you. We have added a figure as per your recommendation (**Fig_1:** Risk factors of non-communicable disease among the respondents (n=249)

**Comment 11:** Discussion, lines 228-234: this section may benefit from some documents and programs of the United Nations High Commissioner for Refugees (UNHCR) for Rohingya refugees in Bangladesh and other relocated countries. Also, expanding this section on the possible implications of the study findings could be beneficial.

**Reply 11:** Thank you for your recommendations. We have added some reference from UNHCR-2017 report on Rohingya refugees in the following lines.

**Line no 211-213:** According to UNHCR 2017 report, the refugee population living in Ukhiya, Cox’sbazar after the exodus of 2017, have an extremely vulnerable mental and physical state due to past experiences of trauma and persecution along with a bleak living situation in the overcrowded camps [19].

**Comment 12:** Declarations, lines 252-253: the referred website for online data repository could not be found with the data used in this study.

**Reply 12:** Thank you. We have updated the link. The data is now available at Mendely data, doi: 10.17632/mckh4gmgtn.1

**Comment 13:** References: the bibliography needs a major revision making the citations uniform using the journal instruction guides for authors.

**Reply 13:**Thank you. We have revised the bibliography according to the journal instruction guides for authors.

**Reviewer 2’s comments to the authors-**

**Comment 1:** The authors describe that interviews were conducted via face-to-face interviews using a semi-structured questionnaire. The description including ‘semi-structured’ and ‘interviews’ implies that qualitative data may have been collected as well. If that is not the case, then the authors should consider rewording the related statements in the methods and abstract sections.

**Reply 1**: Thank you. We appreciate your review of the study questionnaire which was an unintended mistake on our part. We have reworded the related statement in the method and the abstract section.

**Lines 33-34:** Data was collected through face-to-face interviews with a structured questionnaire

**Lines 104-105**: Data collection was conducted from January 2019 to June 2019 through face-to-face interviews using a structured questionnaire.

**Comment 2:** Additionally, the statement ‘Descriptive analysis was done as appropriate for categorical and quantitative variables’ in the methods section of the abstract, should be revised as categorical variables are a type of quantitative variable, and thus the ‘and’ suggests another meaning.

**Reply 2:** Thank you**.** We appreciate your relevant review on the particular statement. We have corrected that part and apologize for the unintended mistake. The corrected statement is given below:

**Lines 35-37:** Descriptive analysis was applied as appropriate for categorical and quantitative variables. Chi-square test and student t test was performed to compare the categories.

**Comment 3:** Authors should include details on how the convenient sampling was conducted in the two camps. Since results show almost equal participation from both genders, did the authors apply any efforts to ensure equitable representation of males and females?

**Reply 3**: Thank you. The issue regarding convenient sampling has been addressed in the following lines of the limitation.

**Lines 282-290:** The sampling method we used for the study is convenient sampling. We conducted the study among refugees from two pre-selected camps who voluntarily agreed to participate in the study. Since the law enforcement authority of government of Bangladesh maintains a strict surveillance system over the refugees we faced difficulty in conducting a broader study over them including every refugee settlement. In addition, we found many of the refugees who experienced various trauma and persecution from the forceful migration not intent enough to participate in the interviews. This is why we preferred convenient sampling as a method.

Also in our study, we have an almost equitable representation of males and females (men 118, women 131) which was completely unintentional and we consider it an opportune situation.

**Comment 4**: Length of residence at the camp is an important factor in this context. Were data on the length of stay at the camp collected? A participant residing at the camp for 3-5 years has been exposed to the NCD-related risk factors for a longer time compared to a resident that has been living there for 6 months-1 year. That is, if the authors presume that living in this specific environment and the related experiences of being forcefully displaced, are the major contributors to the NCD risk factors.

**Reply 4**: Thank you. The two camps we selected for conducting interviews were set up after the latest influx of Rohingya refugees in September 2017 . Therefore, all of the refugees who were living in those camps were living not more than fifteen months from the time of data collection which began in January 2019. Moreover, in our study, we have not seen the association between the living environment or related traumatic experiences of the refugees with the NCD risk factors. We only observed the proportion of the present NCD risk factors among them.

**Comment 5**: Also, were respondents asked about having risk factors prior to the migration? If not, a proxy for this would be information on family history of the NCDs or the risk factors. Was the latter collected? Prior history of NCD or their risk factors can be informative.

**Reply 5**: Thank you for your prudent review. No, the respondents were not asked about having risk factors prior to the migration. Information on family history was not taken as well. We understand that as a limitation of our study.

**Comment 6**: The authors state that ‘the predicted sample size was 241’. The methods used to arrive at the predicted sample size based on the prevalence of NCD risk factors in the Bangladeshi slums, should be identified and somewhat described.

**Reply 6:** Thank you. We have described the issue in the following lines.

**Line 109-113:** A study conducted on among the slum dwellers in Dhaka shows that at least one NCD risk factors was present in 19.5% of participants [20]. Overall prevalence of NCD risk factors were: 36.0%. Based on this prevalence as reference our calculated sample size was 241. Due to the deliberate participation of an adequate number of adult refugees in the study, our final sample size was 249.

**Comment 7:** The use of an existing questionnaire, albeit from the WHO to collect data is a good approach. How was the questionnaire adapted from the WHO STEPs approach to use in this study? Were items omitted etc.? How long was the questionnaire and how long, on average, did it take to conduct the interview?

**Reply 7**: Thank you. In our study, we have followed an adapted questionnaire from STEPS survey done in 2010 which was much briefer than the latest version of the STEPS survey. Considering the vulnerability of the study population we preferred to adopt the briefer version of the STEPS survey which we understand as a limitation of our study. We have discussed the issue in the following lines.

**Lines 114-120:** An adapted (mostly for socio-demographic background) questionnaire for this study was developed using step-I, step-II and, steps III of WHO STEPS protocol based on the 2010 STEPS survey in Bangladesh [14]. Bangla version of the STEPS questionnaire was orally translated into the local Rohingya language by an interpreter during data collection. Although the Rohingya refugees originally live in Mayanmar, the language they speak is similar to Cox’s bazar’s local dialect which assisted the local medical assistants to conduct the interviews.

**Lines 128-135:** Only the core questions about demographic information (step I) with few omissions such as date of birth (due to lack of accuracy on the part of the refugees), marital status etc were used to simplify the questionnaire for the limited time setting. The behavioral components (step I) included the core questions on tobacco, alcohol, physical activity, fruit and vegetable intake and extra salt intake. Information on previous history and treatment of hypertension were also obtained.

**Comment 8**: In the ‘Data analysis’ section, is the reference to ‘quantitative variables’ meant to be ‘continuous variables’? Since the authors also reported means and Std Dev., another analysis besides chi-square test must have been performed to get these results.

**Reply 8**: Thank you. We appreciate your review. We have checked and corrected the issues in the following lines. We have re-analyzed the data and performed chi-square and student t test. However no significant association was found among the variables. We have included these in the data analysis section as well as in the tables in the revised manuscript.

**Lines 156-160:** Descriptive analysis like frequency, percentage, mean and standard deviation were done as appropriate for the categorical variables. Chi-square test and student t-test were performed to see the association among the categories setting theα level at 0.05. 95% confidence interval was done to see the distribution among the population.

**Comment 9:** The authors provide a description and definitions of the variables i.e. BP, BMI, and diabetes. For BP and diabetes, American guidelines are followed. The WHO guidelines (if available for these two measures) would be a more appropriate reference given the context of the study, as done for the BMI.

**Reply 9**: Thank you. We appreciate your suggestions. Unfortunately, there is no WHO guideline available for pre-diabetes from the random blood glucose measurement. As we have only measured random blood sugar, so we used ADA guideline for categorization of RBS. For categorization of blood pressure, we have re-analyzed the data and used JNC 7 guideline which is also adapted by WHO.

**Comment 10:** Since “Blood collection and anthropometric measurement was done by expert medical technologist”, were they, or the persons that conducted the data collection, trained on interviewing procedures? How many persons supported the data collection?

**Reply 10**: Thank you. The medical assistants involved in physical and biochemical measurement were registered medical technologists, and they were also especially trained for this part of data collection. We have mentioned the issues in the following lines.

**Lines 122-124:** Three medical assistants were recruited from Balukhali and Kutupalong health camps and trained for three days for collecting physical and biochemical measurements as well as conducting the interviews**.**

**Comment 11**: For the study limitation, the cross sectional nature of the study.

**Reply 11**: Thank you. We appreciate your review. The issue has been addressed in the following lines.

**Lines 279-282:** The research has some limitations which are worth mentioning. Primarily, we conducted a cross-sectional study to see the proportion of NCD risk factors among the refugees. It was conducted among the adult refugees of selected camp areas in Ukhiya, Cox’s Bazar, which may not represent all the Rohingya refugees who have currently taken shelter in Bangladesh.

**Comment 12 :** Can more advanced analysis i.e. regression be conducted perhaps with ‘number of risk factors ‘as an outcome?

**Reply 12**: Thank you. We appreciate your review. We have already performed bi-variate analysis to see the association between the socio-demographic and NCD risk factors, but regression analysis couldn’t be done due to insignificant p value found in those analyses.

**Comment 13**: Small concerns: grammatical errors

**Reply 13**: Thank you for your review. We have corrected the grammatical errors as much as possible.

**Dear Reviewers, we are grateful for your kind time and substantial review; we believe now the manuscript is more improved, which will satisfy you.**
