# Supplementary material for: Non-communicable diseases risk factors among the forcefully displaced Rohingya population in Bangladesh
Source: PLOS Glob Public Health. 2022 Sep 27;2(9):e0000930. doi: 10.1371/journal.pgph.0000930 (PMC10022334; doi:10.1371/journal.pgph.0000930)
Supplement: Attachment — Submitted filename: Response to reviewers.docx [file pgph.0000930.s003.docx]

Non-communicable diseases risk factors among the forcefully displaced Rohingya population in Bangladesh

PLOS global public health

(Manuscript ID: PGPH-D-22-00771R1)

**Journal Requirements:**

Please review your reference list to ensure that it is complete and correct. If you have cited papers that have been retracted, please include the rationale for doing so in the manuscript text, or remove these references and replace them with relevant current references. Any changes to the reference list should be mentioned in the rebuttal letter that accompanies your revised manuscript. If you need to cite a retracted article, indicate the article’s retracted status in the References list and also include a citation and full reference for the retraction notice.

**Reply:** Thank you. We reviewed the reference list. We added two new references (27 and 28) which have been mentioned below along with the respective line numbers. However, reference no 2,3,9 are available in their respective websites.

**Additional Editor’s comments to the authors**

Thank you for your comments. We have revised the whole manuscript. We have considered and incorporated the revisions you have suggested according to the line numbers as follows:

**Comment 1:** Consider replacing the phrase “is likely to” with “may” in line 217.

**Reply 1:** Thank you. We have followed your advice

**Line 215-216**: The present study indicates that the course of chronic diseases may rise considering the pattern of living and dietary condition of the camp dwellers at Ukhiya.

**Comment 2**. Provide reference for the following statement: “Non communicable diseases have been determined as a significant health challenge among many humanitarian set-up around the world.”

**Reply 2:** Thank you. We have followed your advice

**Line 219-220**: Non communicable diseases have been determined as a significant health challenge among many humanitarian set-up around the world [27].

**Comment 3**: Consider replacing the phrase “the native Bangladeshi population” with “their native Bangladeshi counterparts” in line 234.

**Reply 3:** Thank you. We have followed your advice

**Line 232-233:** Only three out of ten hypertensive patients confirmed taking prescribed anti-hypertensive medicines regularly as opposed to their native Bangladeshi counterparts about half of whom take their anti-hypertensive medications regularly

**Comment 4**: Consider replacing the phrase “lower than the adult Syrian refugees” with “lower than that in adult Syrian refugees” in lines 235-236. Please revise similar grammatical issued throughout the manuscript.

**Reply 4 :** Thank you. We have followed your advice

**Line 233-234:** This percentage was also significantly lower than that in adult Syrian refugees (94.1%) who used to take medications on a regular basis.[22].

**Comment 5:**  Consider replacing “can be” with “is” in line 237.

**Reply 5 :** Thank you. We have followed your advice

**Line 237-240**: Intake of extra salt with food is considered as the precipitating factor for hypertension, and more than one third of the respondents reported of taking extra salt with their meals as a daily habit which was much higher than the Bangladeshi nationals (16.5%).

**Comment 6:** Consider replacing “of taking” with “consuming” in line 239.

**Reply 6 :** Thank you. We have followed your advice

**Line 237-240:** Intake of extra salt with food is considered as the precipitating factor for hypertension, and more than one third of the respondents reported of consuming extra salt with their meals as a daily habit which was much higher than the Bangladeshi nationals (16.5%)

**Comment 7**: Replace “one (14.4%) out of ten” with “14.4%” in line 243.

**Reply 7:** Thank you. We have followed your advice

**Line 241**: 14.4% of the respondents were found to have a pre-diabetic level of random blood sugar.

**Comment 8 :** Replace “percentage of diabetes” with “prevalence of diabetes” in line 247

**Reply 8:** Thank you. We have followed your advice

**Line 244:** it could not appropriately measure the prevalence of diabetes among the participants.

**Comment 9:**  Put the citation [21] after “While only 0.8% of Bangladeshi women are smokers” in line 255-256

**Reply 9 : Line 255-256:** While only 0.8% of Bangladeshi women are smokers [21], around six out of ten Rohingya women have adopted smoking as a regular habit.

**Comment 10:**  Correct the typo “(32.0%)%)” in line 266 and “(1.3%))” in line 269

**Reply 10** : Thank you. The typo is corrected

**Comment 11 :**  Since the study findings are not generalizable across different groups of Rohingya refugees, I recommend reframing the following sentence (lines 301-302), “This study depicts the status of NCD risk factors among the Rohingya refugees in Ukhiya, Cox’s bazar” as follows: “This study depicts the status of NCD risk factors among a group of adult Rohingya refugees in Ukhiya, Cox’s Bazar”.

**Reply 11:** Thank you. We have followed your advice

**Line 297-298** : This study depicts the status of NCD risk factors among a group of adult Rohingya refugees in Ukhiya, Cox’s Bazar”.

**Comment 12**: Change the word “him” in line 309.

**Reply 12:** Thank you. We have followed your advice

**Line 303-305**: Even if the patient is cured, there is a chance of developing a disability consequently, which may become a potential threat of a sizeable economic burden for the government of Bangladesh.

**Comment 13 :** Consider replacing the word “will” with “may” in line 309 and in line 274

**Reply 13:** Thank you. We have followed your advice

**Line 303-305**: Even if the patient is cured, there is a chance of developing a disability consequently, which may become a potential threat of a sizeable economic burden for the government of Bangladesh.

**Line 274-276**: It is presumed that the number of overweight/obesity may rise in the future considering their current condition of living.

**Comment 14:** Give reference on how NCD management could be a sizeable economic burden for the government.

**Reply 14:** Thank you. We added the reference

**Line 303-305**: Even though the patient is cured, there is a chance of developing a disability consequently, which may become a potential threat of a sizeable economic burden for the government of Bangladesh [28]

**Reference:** 28. Bloom DE, Cafiero E, Jané-Llopis E, Abrahams-Gessel S, Bloom LR, Fathima S, Feigl AB, Gaziano T, Hamandi A, Mowafi M, O’Farrell D. The global economic burden of noncommunicable diseases. Program on the Global Demography of Aging; 2012 Jan.

**Comment 15:** Regarding the convenience sample, change the statement “The present study aims to explore the non-communicable disease (NCD) risk factors among the Rohinga population,” in line 98-99 with “The present study aims to explore the non-communicable disease (NCD) risk factors in a convenience sample of the Rohinga refugees.” Consider making similar changes in the abstract as well.

**Reply 15:** Thank you. The changes are made.

**Line 28-29:** The objective of this study was to measure the NCD risk factors among the convenient sample of Rohingya refugees.

**Line 96-97**: The present study aims to explore the non-communicable disease (NCD) risk factors in a convenience sample of the Rohinga refugees.

**Comment 16:**  please add more details than what is currently stated, “Using this prevalence value as reference our calculated sample size was 241.” In particular, provide information on the details of power analysis (e.g., significance level, statistical power, etc.).

**Reply 16:** Thank you. The necessary details are added.

**Line 108- 114**: A study conducted among the slum dwellers in Dhaka shows that at least one NCD risk factors was present in 19.5% of participants [20]. Overall prevalence of NCD risk factors were: 36.0% (95% CI: 31.82–40.41). According to that study p=19.5%, q=(100-19.5)=80.5% at 95% CI, z=1.96 & d=5%. So, sample size n= pqz^2^/d^2^=(19.5x80.5)x(1.96)^2/^5^2^=241.21 Using this prevalence value as reference our calculated sample size was 241. Due to the deliberate participation of an adequate number of adult refugees in the study, our final sample size was 249**.**

**Comment 17**: It is unclear what is meant by “95% confidence interval was done to see the distribution among the population” in line 159-160 – please clarify/ rephrase.

**Reply 17:** Thank you. The necessary rephrasing is done.

**Line 160-161:** 95% confidence interval was done to see the distribution among the population.
